# Effect of Deep Cervical Flexor Muscle Training Using Pressure Biofeedback on Pain and Forward Head Posture in School Teachers with Neck Pain: An Observational Study

**DOI:** 10.1155/2021/5588580

**Published:** 2021-05-22

**Authors:** Ahmad H. Alghadir, Zaheen A. Iqbal

**Affiliations:** ^1^Rehabilitation Research Chair, College of Applied Medical Sciences, King Saud University, Riyadh, Saudi Arabia; ^2^Department of Health and Physical Education, The Education University of Hong Kong, Tai Po, Hong Kong

## Abstract

**Background:**

Teaching is one of the professions where incidence and prevalence of neck pain is high. Prolonged use of computers, which has further increased due to online teaching amid pandemic, is known to cause neck pain and alter posture, while people with forward head posture (FHP) are prone to develop neck pain and related disability. Research has shown that impairment of deep cervical flexor (DCF) muscles leads to insufficiency in coordination, activation, overload, and poor support on cervical structures that further lead to development of neck pain and altered neck posture. The objective of this study was to see the effect of DCF muscle training using pressure biofeedback on pain and FHP in school teachers with neck pain.

**Methods:**

This observational study was conducted at medical center in school premises. Fifty-five school teachers aged between 25 and 40 years with experience of more than 5 years were invited to participate in this study. Subjects were divided in two groups. Both the groups received conventional exercises while in experimental group DCF muscle training using pressure biofeedback was given additionally. Pain and FHP were assessed using NPRS and cranio-vertebral angle using digital photograph technique, respectively, at baseline and end of four weeks of treatment.

**Results:**

Although pain and FHP improved in both the groups, mean improvement in both the measures was more in the group that also received DCF training using pressure biofeedback.

**Conclusions:**

This study shows that although pain and FHP improved following conventional exercises in school teachers with neck pain, mean improvement was more significant among those who received additional DCF muscle training using pressure biofeedback.

## 1. Introduction

Neck pain is one of the most significant work-related health problem that is associated with multiple factors including physical and psychological stress [1, 2]. Teaching is one of the professions where its incidence and prevalence is high [3–5]. Changes from conventional to modern methods of teaching have been identified as the main cause stress among them [6]. Adoption of online teaching methods amid the current pandemic situation, use of computers, laptops, and mobile phones has further increased. Prolonged use of computers leads to adoption of static posture for long duration that causes neck and shoulder pain [2, 7] and has been associated with development of neck and upper limb disorders, including forward head posture (FHP) [8]. It also leads to increased tension in the postural muscles resulting in compressive forces on the spine [9]. Female gender, increasing age, long working hours, history of injury, and frequent head down posture are some of the risk factors that have been associated with development of neck pain among them [10–12].

FHP is a postural disorder, where the head is held forward to the body's center of gravity [13]. At least 66% healthy adults aged between 20 and 50 years were reported to have FHP [14]. It is a common deformity found in patients with cervical headache and cranio-mandibular dysfunction [15–17]. As the head moves forward, load on the neck musculature increases, and neck mobility decreases [18, 19]. FHP has also been associated with neck pain and disability [17, 20]. It is reported to cause neck dysfunction due to mechanical disadvantage of cervical structures, compression of occipital nerves, or triggering of nociceptive afferent in the caudate nucleus [21, 22]. Clinically, FHP can be measured as cranio-vertebral angle (CVA) through lateral photographs [23]. It provides a gross measure of forward positioning of the head on the trunk [24]. Individuals with smaller the CVA are more likely to suffer from neck pain and associated disorders including headache [14, 16, 25].

Deep cervical flexor (DCF) muscles exert their action anterior to the axis of motion of the atlanto-occipital and intervertebral joints that stabilizes the cervical spine during movement [26, 27]. Impairment of these muscles may lead to insufficiency in its coordination, activation, overload, and poor support on cervical structures that can further lead to neck pain and altered neck posture [28–30]. Studies in patients with neck pain have also shown impaired activation and poor endurance of these muscles [1, 15]. Previous studies on DCF muscle training have been shown to decrease neck pain and improve functional disability and ability to maintain upright neutral posture in patients with similar problems [1, 15, 31–33]. It would be interesting to see if DCF training can improve FHP in addition to these effects. The objective of this study was to report the effect of DCF muscle training using pressure biofeedback on FHP and pain among school teachers with neck pain. The research design of this study has been based on similar researches published earlier [1, 33]; however, current study included FHP as the outcome measure. We hypothesize that improvement in pain and FHP would be significantly more after training with pressure biofeedback as compared to the control group.

## 2. Materials and Methods

### 2.1. Subjects

Fifty-five school teachers, aged between 25 and 40 years, who had experience of more than 5 years were invited to participate in this study. They were included if they had neck pain score of 5 or more on numeric pain rating scale (NPRS) and poor endurance of DCF muscles. They were excluded if they had undergone any spinal surgery, any positive neurological sign, or any postural deformity other than FHP. They were informed about aims and nature of this study, and their informed consent was obtained. This study was approved by ethical committee of institutional review board.

### 2.2. Study Design

A pretest, posttest experimental group design was used in the study. Convenience method of sampling was used to select the subjects. They were randomly divided in two groups, control and experimental groups using lottery method. The research design of this study has been based on similar researches published earlier [1, 33].

### 2.3. Procedure

Both the groups received conventional exercises for neck pain. In experimental group, cranio-cervical flexor muscles' training was also done in addition to conventional exercises. Poor endurance of DCF muscles was assessed by performance using cranio-cervical flexion test. Pain and FHP were assessed using NPRS and CVA angle using digital photograph technique, respectively, at baseline (P0 and FHP0) and at the end of four weeks of treatment (P4 and FHP4). Subjects were advised not to take any other treatment for their problem during the study period.

### 2.4. Cranio-Cervical Flexion Test [29, 34]

Subjects were positioned in supine lying, and the air unit of pressure biofeedback (PBU-StabilizerTM, Chattanooga Group, INC., TN) was placed at the posterior aspect of the cervical spine just below the occiput and inflated to a baseline of 20 mmHg. They were instructed to perform the cranio-cervical flexion movement such that the pressure rose to 22 mmHg and hold this position for 10 seconds. A rest of 30 seconds was provided, and the whole procedure was repeated for 24, 26, 28, and 30 mmHg. Final reading was taken when the subject was not able to hold the specific pressure for 10 seconds.

Before the test, subjects were given enough time to practice and examiner observed for any substitution movements during the test. Test was considered poor if subjects could not hold the position at 26 mmHg.

### 2.5. Pain [35]

Pain intensity at the time of testing was measured using NPRS. Subjects were asked to rate their neck pain perception on the 0-10 rating scale, where 0 means no pain while 10 is the worst possible pain.

### 2.6. FHP [36, 37]

FHP was measure using CVA. Subjects were made to stand barefoot near a plumb line fixed to ceiling. Their tragus and seventh cervical (C7) vertebra were marked using plastic pointers. A lateral view digital photograph of each subject was taken that was used to measure CVA formed by the horizontal line passing through C7 and the extending from tragus of the ear to C7, using the digitizing software (Image Tool UTHCSA Version 3.0 university of Texas Health service center, San Antonio, Tx). Smaller angle indicates greater FHP. Reliability of this procedure has been reported to be high [38, 39].

### 2.7. Intervention

Conventional exercises [32, 40] included stretching and strengthening exercises under supervision of a physiotherapist. They comprised of stretching of Sternocleidomastoid, Upper trapezius, Levator scapulae and Pectoralis muscles; and strengthening for cervical flexors, middle and lower Trapezius, and Serratus anterior. Each exercise session included 3 sets of 10 repetitions with 10 seconds hold and rest of 2 minutes in between for 4 weeks.

Cranio-cervical flexor muscle training [1, 41, 42] was done according to protocol prescribed by Jull and Falla et al. Air bag of the pressure biofeedback unit was placed below the occiput, and subjects were asked to perform nodding movement to target 5 pressure levels between 22 and 30 mmHg. Each position is held for 10 seconds. There were 3 sets, 10 repetitions each for four days per week for four weeks. Two minutes rest was given between the sets.

### 2.8. Statistics

Statistical difference was tested using Graph-pad Instat 3.0 Graphpad Software Inc., CA, USA. Normality was analyzed using the Kolmogorov-Smirnov test. Since all the values passed the normality test, parametric tests were used to test the hypothesis in this study. Paired *t*-test was used to compare difference within group. Independent *t*-test was applied to compare the difference between the groups. The difference was considered significant for *p* value <0.05.

## 3. Results

After passing the inclusion and exclusion criteria, 50 subjects (44 female, 6 male) participated in the study. Their baseline characteristics are presented in Table 1. There was no significant difference in characteristics of the subjects between the two groups (*p* > 0.05).

### 3.1. Comparison within the Group

In comparison to baseline, there was significant improvement in neck pain and FHP at the end of 4 weeks (*p* < 0.05) in both the groups (Table 2).

### 3.2. Comparison between the Groups

Intergroup comparison of pain and FHP shows that there was significant difference between the groups (*p* < 0.05). Mean improvement in pain and FHP after four weeks of intervention was more in the experimental group than that in the control group (Table 3).

## 4. Discussion

Although there are various studies that see the effect of DCF training on neck pain, to the best of our knowledge, this is the first study to see the effect of pressure biofeedback on FHP. This study was designed to determine if addition of DCF muscle training using pressure biofeedback to conventional exercises has a better effect on pain and FHP in school teachers with neck pain. Results show that although pain and FHP improved in both the groups, mean improvement in both the measures was more in the group that also received DCF training. Baseline readings of neck pain and FHP in the two groups were not significantly different showing homogenous distribution of subjects.

Since most of the schools around the world have adopted online teaching methods due to current pandemic situation, teachers have been forced to use computers, laptops, and mobile phones for more time during the day. This has further increased the potential to develop psychological stress and musculoskeletal pains throughout the body, especially the neck region. This can precipitate for longer periods if left unchecked. Results of the current study can be applied in such cases with the aim to prevent and treat any associated disorders.

Reduction of pain following conventional as well as pressure biofeedback training can be explained by various mechanisms including increase in endorphins, activation of ergo receptors, and better neuromuscular control after exercises [1, 43, 44].

Literature review suggests that neck disorders are often associated with abnormal neck posture [45]. FHP has been correlated negatively with Northwick Park Questionnaire and NPRS [46]. It has also been shown to predispose temporomandibular disorders [47, 48]. These findings suggest that patients with FHP need urgent attention since they are prone to development of higher pain intensity and disability.

There are two types of external feedbacks that can be provided during exercise, knowledge of result and knowledge of performance [49]. Pressure biofeedback is a noninvasive technique that provides knowledge of performance through the hand held apparatus that can augment the patient's sensory feedback mechanism [1]. It has the capacity of changing the patient's capability of responding as they can control their precise recruitment pattern and help in motor learning [15, 50, 51]. Exercises involving feedback motivate the patient and encourage them to perform better each time [52]. The improvement in muscle performance, flexibility of tight muscles, and increased strength in weak muscles has been attributed to generation of optimal constant torque following pressure biofeedback training [7, 15, 53], which support out results.

Longus colli and capitis are important stabilizers of head on neck posture with major function of maintaining cervical lordosis especially during functional movements [30]. DCF muscles have high density of muscle spindles leading to kinesthetic sense, and their action is influenced by variation of resting head position [15, 40, 54, 55]. Grant et al. have shown that there is reduction in mechano-sensitivity of selected neural, muscular, and articular structures with the improvement in endurance of DCF muscles [56]. Another study has reported that there is improvement in ability to maintain upright cervical posture following DCF training [31]. Improved performance of these muscles could explain improved ability to maintain neutral cervical posture in addition to reduction in perceived neck pain [16, 30, 57].

Development of work-related neck pain is multifactorial [58]. Individual anthropometric variations, postural and mental stress, ergonomically poor furniture, high workload, lack of time for self-care, etc., have been reported to affect school teachers in one or other way [59]. Furthermore, work-related disorders have been reported to prevent teachers from carrying out their normal daily activities, seek medical advice, and even change duties or resign from their job [60, 61]. Such serious outcomes suggest that greater stress should be placed on prevention of such disorders. This can be done through ergonomic education, regular physical exercises, and decreased wok stress. Research like these should focus on developing multidimensional rehabilitation programs at school premises.

### 4.1. Limitations

This study should be repeated on a larger sample size with more diverse cultural representation and causes of neck pain including whiplash injuries. Teachers from different specialties, science, arts, etc., can be included to compare the effect of their subject load on incidence and prevalence of work-related disorders. More such studies are needed to generate a rehabilitation protocol with better base of evidence.

## 5. Conclusion

This study shows that although pain and FHP improved following conventional exercises in school teachers with neck pain, mean improvement was more significant among those who received additional DCF muscle training using pressure biofeedback.

## Figures and Tables

**Table 1:** Baseline characteristics of the subjects: mean ± standard deviation.

	Control group (*n*-25)	Experimental group (*n*-25)	Significance
Age (years)	35.50 ± 2.30	37.45 ± 2.51	ns
Pain (points)	6.47 ± 1.36	7.07 ± 1.49	ns
Cranio-cervical flexion test (mmHg)	22.50 ± 0.75	23.25 ± 0.65	ns
FHP (degrees)	58.66 ± 4.28	56.46 ± 3.96	ns

FHP: forward head posture; ns: not significant.

**Table 2:** Comparison of pain and forward head posture within the groups: mean ± standard deviation.

	Control group (*n*-25)		Significance	Experimental group (*n*-25)		Significance
	Preintervention	Postintervention		Preintervention	Postintervention	
Pain (points)	6.47 ± 1.36	4.36 ± 1.50	^∗^	7.07 ± 1.49	4.89 ± 1.23	^∗^
FHP (degrees)	58.66 ± 4.28	60.36 ± 3.95	^∗^	56.46 ± 3.96	59.56 ± 3.90	^∗^

FHP: forward head posture; ^∗^*p* < 0.05.

**Table 3:** Comparison of differences in the means between the groups from postintervention to preintervention of pain and forward head posture.

	Mean change postintervention		Significance
	Control group	Experimental group	
Pain (points)	−2.11 ± 1.25	−2.18 ± 1.35	^∗^
FHP (degrees)	1.70 ± 3.90	3.1 ± 3.86	^∗^

FHP: forward head posture; ^∗^*p* < 0.05.

## Data Availability

The datasets used in this study are available from the corresponding author on reasonable request.

## Abbreviations

FHP: Forward head posture
CV: Cranio-vertebral angle
DCF: Deep cervical flexor
NPRS: Numerical pain rating scale.

## References

[1] Iqbal Z. A., Rajan R., Khan S. A., Alghadir A. H. (2013). Effect of deep cervical flexor muscles training using pressure biofeedback on pain and disability of school teachers with neck pain. *Journal of Physical Therapy Science*.

[2] Ariens G. A., Bongers P. M., Douwes M. (2001). Are neck flexion, neck rotation, and sitting at work risk factors for neck pain? Results of a prospective cohort study. *Occupational and Environmental Medicine*.

[3] Nikolovski D., Saric-Tanaskovic M. (2006). Risk factors and health condition of workers in school environment. *Srpski arhiv za Celokupno Lekarstvo*.

[4] Fjellman-Wiklund A., Brulin C., Sundelin G. (2003). Physical and psychosocial work-related risk factors associated with neck-shoulder discomfort in male and female music teachers. *Medical Problems of Performing Artists*.

[5] Cardoso J. P., Ribeiro I. D., Araújo T. M., Carvalho F. M., Reis E. J. (2009). Prevalence of musculoskeletal pain among teachers. *Revista Brasileira de Epidemiologia*.

[6] Bergqvist U., Wolgast E., Nilsson B., Voss M. (1995). Musculoskeletal disorders among visual display terminal workers: individual, ergonomic, and work organizational factors. *Ergonomics*.

[7] Hunting W., Laubli T., Grandjean E. (1981). Postural and visual loads at VDT workplaces. I. Constrained postures. *Ergonomics*.

[8] Sterling M., Jull G., Vicenzino B., Kenardy J., Darnell R. (2003). Development of motor system dysfunction following whiplash injury. *Pain*.

[9] Harreby M., Neergaard K., Hesselsoe G., Kjer J. (1995). Are radiologic changes in the thoracic and lumbar spine of adolescents risk factors for low back pain in adults? A 25-year prospective cohort study of 640 school children. *Spine (Phila Pa 1976)*.

[10] Kurumatani N., Iki M., Katagi K. (1984). A study on occupational cervicobrachial disorder (OCD) of nursery school teachers based on subjective symptoms related to OCD. *Sangyo Igaku*.

[11] Chiu T. T., Lam P. K. (2007). The prevalence of and risk factors for neck pain and upper limb pain among secondary school teachers in Hong Kong. *Journal of Occupational Rehabilitation*.

[12] Erick P. N., Smith D. R. (2014). The prevalence and risk factors for musculoskeletal disorders among school teachers in Botswana. *Occupational Medicine & Health Affairs*.

[13] Rizo A. M. H., Pascual-Vaca Á. O., Cabello M. A., Blanco C. R., Pozo F. P., Carrasco A. L. (2012). Immediate effects of the suboccipital muscle inhibition technique in craniocervical posture and greater occipital nerve mechanosensitivity in subjects with a history of orthodontia use: a randomized trial. *Journal of Manipulative and Physiological Therapeutics*.

[14] Griegel-Morris P., Larson K., Mueller-Klaus K., Oatis C. A. (1992). Incidence of common postural abnormalities in the cervical, shoulder, and thoracic regions and their association with pain in two age groups of healthy subjects. *Physical Therapy*.

[15] Nezamuddin M., Anwer S., Khan S. A., Equebal A. (2013). Efficacy of pressure-biofeedback guided deep cervical flexor training on neck pain and muscle performance in visual display terminal operators. *Journal of Musculoskeletal Research*.

[16] Watson D. H., Trott P. H. (1993). Cervical headache: an investigation of natural head posture and upper cervical flexor muscle performance. *Cephalalgia*.

[17] Yip C. H. T., Chiu T. T. W., Poon A. T. K. (2008). The relationship between head posture and severity and disability of patients with neck pain. *Manual Therapy*.

[18] Padrós E. (2004). Cómo cuantificar las funciones y la postura en la consulta de ortodoncia. *Ortodoncia Clínica*.

[19] De-La-Llave-Rincón A. I., Fernández-De-Las-PeÑas C., Palacios-CeÑa D., Cleland J. A. (2009). Increased forward head posture and restricted cervical range of motion in patients with carpal tunnel syndrome. *Journal of Orthopaedic & Sports Physical Therapy*.

[20] Silva A. G., Punt T. D., Sharples P., Vilas-Boas J. P., Johnson M. I. (2009). Head posture and neck pain of chronic nontraumatic origin: a comparison between patients and pain-free persons. *Archives of Physical Medicine and Rehabilitation*.

[21] Grimmer-Somers K., Milanese S., Louw Q. (2008). Measurement of cervical posture in the sagittal plane. *Journal of Manipulative and Physiological Therapeutics*.

[22] Pilat A. (2003). *Terapias Miofasciales: Inducción Miofascial*.

[23] Sonnesen L., Bakke M., Solow B. (2001). Temporomandibular disorders in relation to craniofacial dimensions, head posture and bite force in children selected for orthodontic treatment. *The European Journal of Orthodontics*.

[24] Gandevia S. C., Phegan C. M. (1999). Perceptual distortions of the human body image produced by local anaesthesia, pain and cutaneous stimulation. *The Journal of Physiology*.

[25] Grimmer K. A., Williams M. T., Gill T. K. (1999). The associations between adolescent head-on-neck posture, backpack weight, and anthropometric features. *Spine (Phila Pa 1976)*.

[26] Painkra J. P., Kumar S., Anwer S., Kumar R., Nezamuddin M., Equebal A. (2014). Reliability of an assessment of deep neck flexor muscle endurance test: a cross-sectional study. *International Journal of Therapy and Rehabilitation*.

[27] Mayoux-Benhamou M., Revel M., Vallee C., Roudier R., Barbet J., Bargy F. (1994). Longus colli has a postural function on cervical curvature. *Surgical and Radiologic Anatomy*.

[28] Falla D., Jull G., Hodges P. W. (2004). Feedforward activity of the cervical flexor muscles during voluntary arm movements is delayed in chronic neck pain. *Experimental Brain Research*.

[29] Falla D. L., Jull G. A., Hodges P. W. (2004). Patients with neck pain demonstrate reduced electromyographic activity of the deep cervical flexor muscles during performance of the craniocervical flexion test. *Spine (Phila Pa 1976)*.

[30] Falla D. (2004). Unravelling the complexity of muscle impairment in chronic neck pain. *Manual Therapy*.

[31] Falla D., Jull G., Russell T., Vicenzino B., Hodges P. (2007). Effect of neck exercise on sitting posture in patients with chronic neck pain. *Physical Therapy*.

[32] Nezamuddin M., Khan S. A., Hameed U. A., Anwer S., Equebal A. (2013). Efficacy of pressure biofeedback guided deep cervical flexor training on forward head posture in visual display terminal operators. *Indian Journal of Physiotherapy and Occupational Therapy - An International Journal*.

[33] Iqbal Z. A., Alghadir A. H., Anwer S. (2021). Efficacy of deep cervical flexor muscle training on neck pain, functional disability, and muscle endurance in school teachers: a clinical trial. *BioMed Research International*.

[34] Jull G. A., O'Leary S. P., Falla D. L. (2008). Clinical assessment of the deep cervical flexor muscles: the craniocervical flexion test. *Journal of Manipulative and Physiological Therapeutics*.

[35] Cleland J. A., Childs J. D., Whitman J. M. (2008). Psychometric properties of the Neck Disability Index and Numeric Pain Rating Scale in patients with mechanical neck pain. *Archives of Physical Medicine and Rehabilitation*.

[36] Braun B. L., Amundson L. R. (1989). Quantitative assessment of head and shoulder posture. *Archives of Physical Medicine and Rehabilitation*.

[37] Fernandez-de-Las-Penas C., Alonso-Blanco C., Cuadrado M., Pareja J. (2006). Forward head posture and neck mobility in chronic tension-type headache: a blinded, controlled study. *Cephalalgia*.

[38] Raine S., Twomey L. T. (1997). Head and shoulder posture variations in 160 asymptomatic women and men. *Archives of Physical Medicine and Rehabilitation*.

[39] Kerry C. (2003). Reliability of measuring natural head posture using the craniovertebral angle. *Irish Ergonomics Review*.

[40] Kendall F. P., EK M. C., Kendall H. O. (1983). *Muscles, Testing and Function: Testing and Function*.

[41] Jull G., Barrett C., Magee R., Ho P. (1999). Further clinical clarification of the muscle dysfunction in cervical headache. *Cephalalgia*.

[42] Falla D., Jull G., Hodges P., Vicenzino B. (2006). An endurance-strength training regime is effective in reducing myoelectric manifestations of cervical flexor muscle fatigue in females with chronic neck pain. *Clinical Neurophysiology*.

[43] Thorén P., Floras J. S., Hoffmann P., Seals D. R. (1990). Endorphins and exercise. *Medicine & Science in Sports & Exercise*.

[44] Hutton R. S., Atwater S. W. (1992). Acute and chronic adaptations of muscle proprioceptors in response to increased use. *Sports Medicine*.

[45] Osmotherly P., Attia J. (2008). The interplay of static and dynamic postural factors in neck pain. *Hong Kong Physiotherapy Journal*.

[46] Lau M., Chiu T. T. W., Lam T.-H. (2010). Measurement of craniovertebral angle with electronic head posture instrument: criterion validity. *Journal of Rehabilitation Research and Development*.

[47] Strini P. J. S. A., de Godoi Machado N. A., Gorreri M. C., de Freitas Ferreira A., da Cunha Sousa G., Neto A. J. F. (2009). Postural evaluation of patients with temporomandibular disorders under use of occlusal splints. *Journal of Applied Oral Science*.

[48] Matheus R. A., de Moraes Ramos-Perez F. M., Menezes A. V. (2009). The relationship between temporomandibular dysfunction and head and cervical posture. *Journal of Applied Oral Science*.

[49] Winstein C. J. (1991). Knowledge of results and motor learning—implications for physical therapy. *Physical Therapy*.

[50] O'Sullivan S. B., Schmitz T. J., Fulk G. (2013). *Physical Rehabilitation*.

[51] Huang H., Wolf S. L., He J. (2006). Recent developments in biofeedback for neuromotor rehabilitation. *Journal of Neuroengineering and Rehabilitation*.

[52] Kluger A. N., DeNisi A. (1996). *The effects of feedback interventions on performance: a historical review, a meta-analysis, and a preliminary feedback intervention theory*.

[53] Ballantyne F., Fryer G., McLaughlin P. (2003). The effect of muscle energy technique on hamstring extensibility: the mechanism of altered flexibility. *Journal of Osteopathic Medicine*.

[54] Boyd-Clark L., Briggs C., Galea M. (2002). Muscle spindle distribution, morphology, and density in longus colli and multifidus muscles of the cervical spine. *Spine (Phila Pa 1976)*.

[55] Jull G., Falla D., Treleaven J., Hodges P., Vicenzino B. (2007). Retraining cervical joint position sense: the effect of two exercise regimes. *Journal of Orthopaedic Research*.

[56] Grant R., Jull G., Spencer T. (1997). Active stabilisation training for screen based keyboard operators - a single case study. *Australian Journal of Physiotherapy*.

[57] Ylinen J., Ruuska J. (1994). Clinical use of neck isometric strength measurement in rehabilitation. *Archives of Physical Medicine and Rehabilitation*.

[58] Ono Y., Imaeda T., Shimaoka M. (2002). Associations of length of employment and working conditions with neck, shoulder and arm pain among nursery school teachers. *Industrial Health*.

[59] Grimes P., Legg S. (2004). Musculoskeletal disorders (MSD) in school students as a risk factor for adult MSD: a review of the multiple factors affecting posture, comfort and health in classroom environments. *Journal of the Human-Environment System*.

[60] Erick P. N., Smith D. R. (2011). A systematic review of musculoskeletal disorders among school teachers. *BMC Musculoskeletal Disorders*.

[61] Chong E. Y., Chan A. H. (2010). Subjective health complaints of teachers from primary and secondary schools in Hong Kong. *International Journal of Occupational Safety and Ergonomics*.

